# Natural and non-natural antioxidative compounds: potential candidates for treatment of vascular calcification

**DOI:** 10.1038/s41420-019-0225-z

**Published:** 2019-11-13

**Authors:** Chia-Ter Chao, Hsiang-Yuan Yeh, You-Tien Tsai, Pei-Huan Chuang, Tzu-Hang Yuan, Jenq-Wen Huang, Huei-Wen Chen

**Affiliations:** 1 Department of Medicine, National Taiwan University Hospital BeiHu Branch, College of Medicine, National Taiwan University, Taipei, Taiwan; 2 Department of Internal Medicine, National Taiwan University College of Medicine, Taipei, Taiwan; 3 Graduate Institute of Toxicology, National Taiwan University College of Medicine, Taipei, Taiwan; 4 School of Big Data Management, Soochow University, Taipei, Taiwan; 5 Nephrology Division, Department of Internal Medicine, National Taiwan University Hospital, Taipei, Taiwan

**Keywords:** Calcification, Cell biology

## Abstract

Vascular calcification (VC) is highly prevalent in patients with advanced age, or those with chronic kidney disease and diabetes, accounting for substantial global cardiovascular burden. The pathophysiology of VC involves active mineral deposition by transdifferentiated vascular smooth muscle cells exhibiting osteoblast-like behavior, building upon cores with or without apoptotic bodies. Oxidative stress drives the progression of the cellular phenotypic switch and calcium deposition in the vascular wall. In this review, we discuss potential compounds that shield these cells from the detrimental influences of reactive oxygen species as promising treatment options for VC. A comprehensive summary of the current literature regarding antioxidants for VC is important, as no effective therapy is currently available for this disease. We systematically searched through the existing literature to identify original articles investigating traditional antioxidants and novel compounds with antioxidant properties with regard to their effectiveness against VC in experimental or clinical settings. We uncovered 36 compounds with antioxidant properties against VC pathology, involving mechanisms such as suppression of NADPH oxidase, BMP-2, and Wnt/β-catenin; anti-inflammation; and activation of Nrf2 pathways. Only two compounds have been tested clinically. These findings suggest that a considerable opportunity exists to harness these antioxidants for therapeutic use for VC. In order to achieve this goal, more translational studies are needed.

## Facts

1. Anti-oxidants are compounds that counteract oxidative stress within cellular microenvironments, divided broadly into those of natural and non-natural origin.
2. Reactive oxygen species are vital pathogenic players in atherosclerotic cardiovascular diseases, against which anti-oxidants can be excellent sources of treatment candidates.
3. Vascular calcification (VC) contributes significantly to cardiovascular morbidity and mortality in at-risk population including those with diabetes and kidney diseases, but effective therapies for VC remain in question.

## Open questions

1. To what extent anti-oxidative therapies have been utilized to treat VC experimentally and clinically?
2. What molecular mechanisms are involved in the observed therapeutic efficacy of antioxidants against VC?
3. How to choose the optimal dose and duration of different anti-oxidants for the treatment of VC in light of the existing literature?

## Introduction

Cardiovascular diseases are the leading cause of mortality worldwide, accounting for nearly one-fourth of total deaths globally, while real-world data suggest that the burden is likely higher than we deduce based on trial information^1^. Despite the substantial benefit conferred by the widespread use of statin for cholesterol reduction as well as the promotion of guideline adherence, the global mortality due to cardiovascular diseases continued to increase from the 1990s to 2013 (ref. ^2^). This trend has been partly attributed to population aging, but risk factors other than demographic influences likely contribute as well, such as atypical factors including chronic inflammation, mineral dysregulation, and excess pathogenic hormonal milieu^3^. Findings from previous studies suggested vascular calcification (VC) as an important phenotype arising from the influences of atypical cardiovascular risk factors^4^. Indeed, VC is highly prevalent in certain patient populations, including those with chronic kidney disease (CKD) or diabetes mellitus, and the presence of VC is predictive of an increased risk of cardiovascular diseases, overall mortality, and hospitalization independent of multiple confounders^5^. This detrimental influence of VC is mainly related to enhanced arterial stiffness, ventricular pressure load with hypertrophy and coronary ischemia. However, effective therapies for VC remain scant, and even the promising ones involve the optimization of divalent ions and interplay between hormones within the scope of mineral bone disorder without proven efficacy^6^. Consequently, novel therapies that tackle alternative aspects of VC pathophysiology are urgently needed.

## The central role of oxidative stress in VC

The pathophysiology of VC is complex, but most agree that active osteoid matrix-like deposition by transdifferentiated vascular smooth muscle cells (VSMCs) or infrequently by other vascular wall constituent cells plays an important role. This highly regulated cellular phenotypic switch, accompanied by the loss of contractile function (for VSMCs) and the gain of synthetic function, can be stimulated by disturbed mineral homeostasis (especially a high phosphate environment), hyperglycemia with advanced glycation endproduct (AGE) accumulation, or inflammatory cytokines, which coincide with the clinical features of the high-risk population for developing VC^7^. The process of active mineralization is further augmented by the loss of calcification inhibitors such as matrix Gla protein (MGP), pyrophosphate, or fetuin-A^8^.

A core component that drives the phenotypic change of VSMCs during VC is believed to be premature vascular aging and VSMC senescence^9^. Oxidative stress (OS), manifested by the production of reactive oxygen species (ROS) and counteracted by antioxidant mechanisms, is a major inducer of cellular senescence. OS presumably induces damages to vital cellular substructures including DNA, protein, and membrane/cytoplasmic lipids. The sources of ROS in calcified VSMCs include cellular enzymes such as xanthine oxidase, nicotinamide adenine dinucleotide phosphate oxidase (NADPH oxidase, Nox), and the cytochrome P450 systems. The downstream events of redox imbalance may include telomere shortening, mitochondrial DNA damage, and direct mutagenic influences on gene fragments involved in contractile functions, thereby predisposing VSMCs to aging and the loss of smooth muscle cell phenotypes. These events are followed by osteogenic transcription factor activation^10,11^. In addition, mitochondrial damage can be accompanied by further leakage of ROS from inner mitochondrial membrane, perpetuating a vicious cycle of ROS accumulation and a greater degree of apoptosis. Inflammation following cytokine release triggered by OS can further set the stage for VC development.

In light of the importance of ROS in the pathogenesis of VC, it is expected that antioxidants can serve as a promising therapeutic approach for managing VC, a disorder currently without effective treatments^12^. We thus performed a literature-based review to summarize the existing knowledge with regard to the utility of antioxidants against VC, in experimental models and in clinical studies.

## Strategy of literature search

**Fig. 1:**
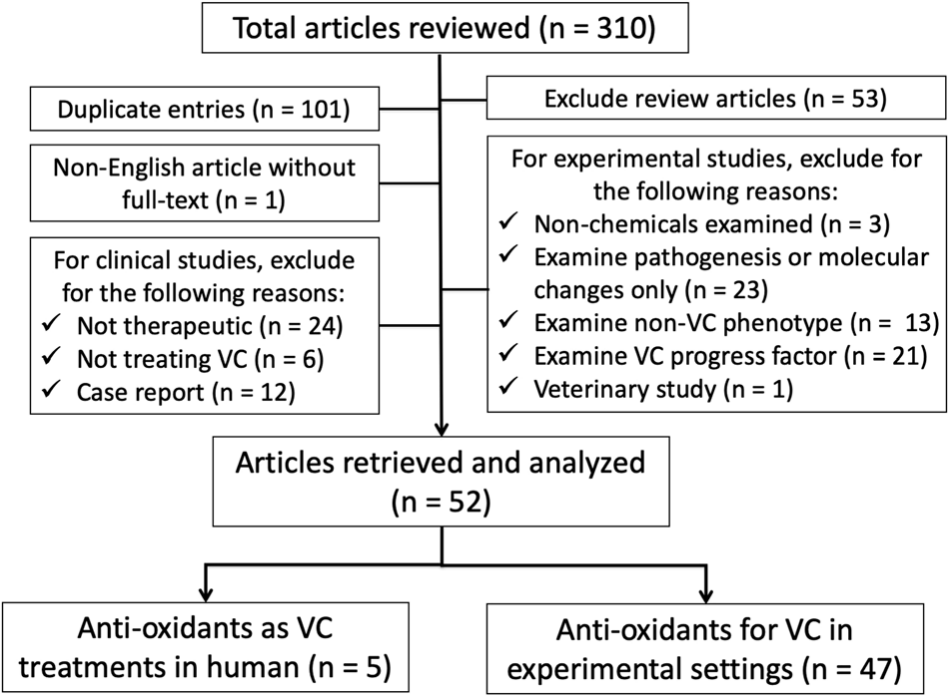
The algorithm of study retrieval from the literature and the application of selection criteria. VC vascular calcification

We systematically searched through the existing literature, using the following combinations of keywords and MESH terms: “vascular smooth muscle cells”, “calcification”, or “vascular calcification” and “reactive oxygen species”, “oxidative stress”, “antioxidant”, or “anti-oxidant” in the PUBMED and MEDLINE databases up to 7 June 2019 to retrieve relevant articles for analysis; 310 articles were identified, of which 101 were excluded as duplicate entries (Fig. 1). We subsequently reviewed the abstracts of the remaining 209 articles in depth, and excluded review articles (*n* = 53) and those for which the full text was unavailable (*n* = 1), after which these articles were classified into clinical (*n* = 47) and experimental (*n* = 108) research studies. For clinically oriented studies, we further excluded those whose focuses were not therapeutic, therapeutic but not against VC, or case reports; for experimental ones, we excluded those not examining chemicals, examining pathogenesis only, examining non-VC pathologies, or those examining aggravating factors (Fig. 1).

**Fig. 2:**
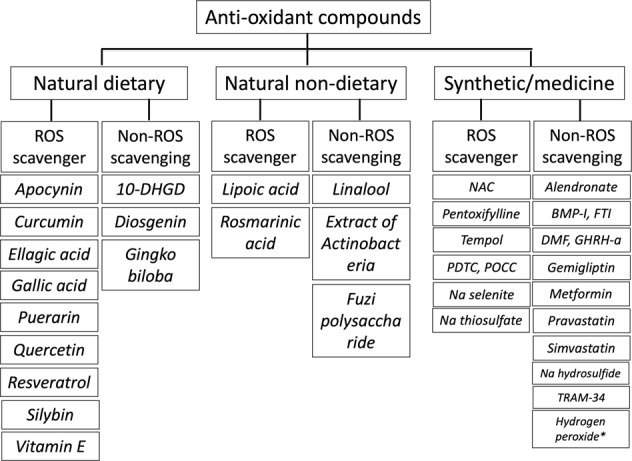
The proportion of identified antioxidant compounds within each category, stratified based on the presence or absence of ROS scavenging ability. 10-DHGD 10-dehydrogingerdione, BMP-I bone morphogenetic protein inhibitor, DMF dimethylfumarate, FTI farnesyl transferase inhibitor, GHRH-a growth hormone-releasing hormone receptor agonist, NAC *N*-acetylcysteine, PDTC pyrrolidine dithiocarbamate, POCC poly(1,8-octamethylene-citrate-co-cysteine), ROS reactive oxygen species. * Very low dose (0.01 mM)

The remaining 52 articles with their full-texts were finally reviewed with data extracted for description. Among these, 5 (9.6%) involved clinical studies that examined antioxidants for treatment of VC, while 47 (90.4%) involved experimental studies. For clinical therapeutic studies, four (80%) used parenteral sodium thiosulfate (STS), while one (20%) used topical cerium nitrate^13–17^. For experimental approaches, totally 36 compounds with antioxidant property have been tested against VC, including 12 natural dietary substances, 5 natural non-dietary substances, and 19 pharmaceuticals or synthetic chemicals (Fig. 2)^18–64^. Among compounds from each category, they are further divided into those with and without ROS scavenging ability.

We examined candidate antioxidants with regard to their effects on VC and the potential mechanisms involved if the articles used experimental approaches, and the antioxidants that have been tested for therapeutic efficacy against VC in clinical settings, in the following sections. Compounds which have been tested in animal models are tabulated separately to outline the dosage and models through which anti-calcification effect are shown separately (Table 1).

**Table 1:** Summary of animal studies investigating the effect of anti-oxidants on vascular calcification

VC models	Animal	Antioxidants	Doses	Effect	Reference
Adenine-induced CKD	Rat	Sodium thiosulfate	0.4 g/kg thrice weekly IP	Reduced aortic calcification, serum Ca^2+^, induced metabolic acidosis and calciuria	^36^
High cholesterol diet + VitD	Rabbit	Lipoic acid	120 µmol/kg/day PO	Decrease AV calcification	^22^
ApoE^−/−^ with nephrectomy	Mouse	Simvastatin	100 mg/kg/day PO	Decrease aortic plaque calcification; neutral serum cholesterol levels	^32^
LDLR^−/−^ + high fat diet	Mouse	BMP antagonist	2.5 mg/kg/day IP	Reduced aortic atheroma, calcification, and cholesterol levels	^39^
Adenine-induced CKD	Rat	Tempol	3 mM in drinking water PO	Reduced aortic calcification; neutral serum Ca^2+^/PO_4_^−^, vitamin D, PTH levels	^40^
High dose VitD	Mouse	Lipoic acid	40 mg/kg/day IP	Reduced aortic calcification, apoptosis, and improved mitochondrial function	^23^
ApoE^−/−^ + partial renal ablation	Mouse	Farnesyltransferase inhibitor	50 mg/kg/day IP	Reduced aortic atheroma, calcification, and plaque collagen amount	^41^
Warfarin-induced VC	Rat	Quercetin	10 mg/kg/day PO	Reduced aortic calcification and lower SBP/PP	^26^
MGP^−/−^	Mouse	Quercetin	0.02% in drinking water PO	Reduced aortic calcification	^28^
Adenine-induced CKD	Rat	Diosgenin	10–40 mg/kg/day PO	Reduced aortic calcification; neutral serum Ca^2+^/PO_4_^−^, uric acid, creatinine levels	^20^
High fat diet + STZ + warfarin	Rat	Apocynin	2.5 mg/kg/day PO	Reduced femoral artery calcification and tissue AGEs	^18^
High cholesterol diet + VitD	Rabbit	Lipoic acid	0.12 mmol/kg/day PO	Reduced aortic calcification, stiffness, and preserved vasoconstrictor responses	^24^
Adenine-induced CKD	Rat	Diosgenin	40 mg/kg/day PO	Reduced aortic RUNX2 expression and decreased coronary resistance	^21^
Zucker obese animals + 5/6 nephrectomy + VitD	Rat	Vitamin E	30,000 mg/kg PO	Reduced aortic calcification	^35^
High fat diet + 5/6 nephrectomy	Rat	Vitamin E	30,000 mg/kg PO	Reduced aortic, gastric, and pulmonary calcium content	^55^
Adenine-induced CKD	Rat	Quercetin	25 mg/kg/day PO	Reduced aortic calcification and lower phosphate, creatinine, and uric acid levels	^29^
l-NAME-induced hypertension	Rat	Ellagic acid	10–30 mg/kg/day PO	Reduced aortic calcification, aortic thickness, SBP, and restored vasoconstrictor/dilator responses	^56^
Adenine-induced CKD	Rat	Gemigliptin	10–20 mg/kg/day IP	Reduced aortic calcification	^57^
Adenine-induced CKD	Rat	Quercetin	100 mg/kg/day PO	Reduced aortic calcification and restore aortic mitochondrial integrity	^30^
OPG^−/−^	Mouse	GHRH agonist	5 µg/kg/day SC	Reduced aortic calcification	^58^
Adenine-induced CKD	Rat	Linalool	100–150 mg/kg/day PO	Reduced aortic calcification, aortic ROS scavenging	^61^
5/6 nephrectomy + high Ca/P diet + VitD	Rat	Puerarin	400 mg/kg/day PO	Reduced aortic calcification and lower inflammatory cytokines	^62^
High cholesterol diet	Rabbit	10-DHGD, Pentoxifylline	10-DHGD (10 mg/kg/day PO) Pentoxifylline (40 mg/kg/day PO)	Reduced aortic calcification, lower serum total/LDL/HDL cholesterol, triglyceride	^64^

*10-DHGD* 10-dehydrogingerdione, *AGE* advanced glycation endproducts, *AV* aortic valve, *BMP* bone morphogenic factor, *CKD* chronic kidney disease, *GHRH* growth hormone-releasing hormone, *HDL* high-density lipoprotein, *IP* intraperitoneal, *LDLR* low-density lipoprotein receptor, *l-NAME* *N*(ω)-nitro-l-arginine methyl ester, *MGP* matrix Gla protein, *OPG* osteoprotegerin, *PO* per oral, *PP* pulse pressure, *PTH* parathyroid hormone, *ROS* reactive oxygen species, *SBP* systolic blood pressure, *SC* subcutaneous, *STZ* streptozotocin, *VC* vascular calcification, *VitD* vitamin D

## Antioxidants from natural and dietary sources for treatment of VC

To date, in vitro and in vivo experiments have tested 12 natural antioxidants that are also dietary ingredients, including 10-dehydrogingerdione (10-DHGD)^64^, apocynin^18,19^, curcumin^38^, diosgenin^20,21^, ellagic acid^56^, gallic acid^48^, gingko biloba extracts^50^, puerarin^62^, quercetin^25–31^, resveratrol^52^, silybin^38^, and vitamin E^34,35^. Among these compounds, apocynin, diosgenin, quercetin, and vitamin E have been shown by multiple studies to have anti-VC properties. Nine (75%) of these compounds possess ROS scavenging ability, while others do not (Fig. 2).

Apocynin is isolated from the plant *Apocynum*, also known as dogbane, and has been used as a herbal medicine by Native Americans and as herbal tea in several Asian countries^65^. Through inhibition of Nox, apocynin has been found to reduce superoxide generation and ameliorate inflammation in various animal models^66^. Brodeur et al. found that the administration of apocynin to streptozotocin-induced diabetic rats with warfarin-induced arterial calcification could substantially improve the severity of femoral artery VC, although the mechanism involved has not been tested^18^. Other studies further disclosed that apocynin ameliorated VC by suppressing bone morphogenetic protein-2 (BMP-2) expression and attenuating the phosphorylation of ERK1/2, thereby reducing RUNX2 and osteopontin levels based on an angiotensin II-induced osteogenic transformed VSMCs model^19^. Diosgenin is a phytosteroidal saponin purified from fenugreek (*Trigonella foenum-graecum*) seeds and can be biosynthesized from cholesterol. Previous studies demonstrated that diosgenin possessed lipid and glucose-lowering effects in addition to anti-inflammatory and antioxidant properties^67^. Manivannan et al. found that diosgenin could reduce aortic calcification in CKD rats in a dose-dependent manner by increasing antioxidant enzyme levels, lowering lipid peroxidation^20^, and stimulating endothelial nitric oxide synthase (eNOS) activities, thereby improving coronary reserves^21^. Vitamin E, including lipophilic tocopherols and tocotrienols, is universally found in green vegetables, nuts, eggs, and milk, and has long been known to exhibit strong antioxidant properties. Mody et al., in a pilot study nearly two decades ago, found that a vitamin E analog could halt the osteoblastic differentiation of aortic VSMCs induced by oxidized low-density lipoprotein (ox-LDL)^34^. Vitamin E has subsequently been shown to restore plasma antioxidant levels, reduce AGEs, and suppress aortic calcification severity in obese CKD rats and in human VSMCs^35^.

Quercetin, a naturally occurring flavanol and a secondary plant metabolite, is enriched in many fruits and vegetables and is also used as a dietary supplement^68^. It has been extensively investigated with respect to its effect against VC based on our literature search results. Lu et al. disclosed that quercetin abolished heat shock protein 72 induced anti-calcification effect in vitro and ex vivo^25^; however, another group later found that quercetin completely nullified warfarin-induced aortic calcification and aortic medial cartilaginous metaplasia by inhibiting transglutaminase 2 and β-catenin expression in vitro^26–28^. Alternatively, quercetin could reduce aortic calcification in CKD rats partially by increasing superoxide dismutase 2 (SOD2) levels and modulating the inducible NO synthase (iNOS)/MAPK pathway^29^. Cui et al. further showed that quercetin restored mitochondrial integrity with improved membrane potential, ATP production, reduced fragmentation/fission, attenuated Drp1 phosphorylation, and reversed the apoptotic process in calcified VSMCs^30^. Recently, Liang et al. showed that quercetin might be protective for VSMCs by attenuating the expression of BMP-2 and toll-like receptor (TLR)−4 in ox-LDL-induced calcification^31^.

On the other hand, the antioxidant properties of 10-DHGD, curcumin, ellagic acid, gallic acid, gingko biloba, puerarin, resveratrol, and silybin were described in single reports only. 10-DHGD is a biologically active component extracted from the rhizomes of ginger (*Zingiber officinale*), a medicinal plant that is also used as dietary spices^69^. 10-DHGD has been shown to exhibit anti-inflammatory and antioxidant properties, and to stimulate NO release^70^. Elseweidy et al. revealed that 10-DHGD could attenuate the development of aortic atherosclerotic calcifications in dyslipidemic rabbits by downregulating the expression of aortic BMP-2, Wnt3a, tumor necrosis factor-α (TNF-α), receptor activator of nuclear factor-κB (RANK), and RUNX2^64^. Curcumin is a polyphenol extracted from the dried Rhizomes of *Curcuma longa* and spices such as turmeric. It is known to possess anti-inflammatory and antioxidant properties^71^. Silybin is the main active ingredient of *Silybum marianum* extracts. It exhibits antioxidant property and is mostly used for treating viral hepatitis^72^. Roman-Garcia et al. showed that curcumin and silybin reduced ROS levels and calcification of VSMCs^38^. Being a natural polyphenol with antioxidant property, gallic acid and its derivative, ellagic acid, mainly exist in fruits such as pomegranates, grapes, berries, and tea leaves^73^. Gallic acid has been shown to reduce osteoblastic differentiation of VSMCs by suppressing BMP-2/Smad 1/5/8 signaling, while ellagic acid has been shown to decrease calcium deposition in aortic walls and improves blood pressure in hypertensive rats^48,56^. *Ginkgo biloba* extracts are mixtures containing several flavanols, ginkgolides, and bilobalides, and are found to be vasoactive and antioxidative^74^. Li et al. demonstrated that *Ginkgo biloba* could attenuate VSMC calcification by downregulating NF-κB and reducing ROS levels^50^. The primary extract of *Pueraria lobata*, puerarin, is used as an herbal medicine for treating acute ischemic stroke. Its active ingredient is an isoflavone glycoside^75^. Liu et al. disclosed that puerarin inhibited ROS production, reduced NF-κB and BMP-2 expression, and IL-1β levels, resulting in reduced aortic calcification^62^. Finally, resveratrol, a phytoalexin and polyphenol mainly contained in plants such as grapes and its derivatives such as red wine, has been repeatedly shown to be vasculo- and cardio-protective^76^. Zhang et al. found that resveratrol reduced VSMC calcification by activating Nrf2 and Sirt1, upregulating Klotho expression and inhibiting fibroblast growth factor-23 (ref. ^52^).

**Fig. 3:**
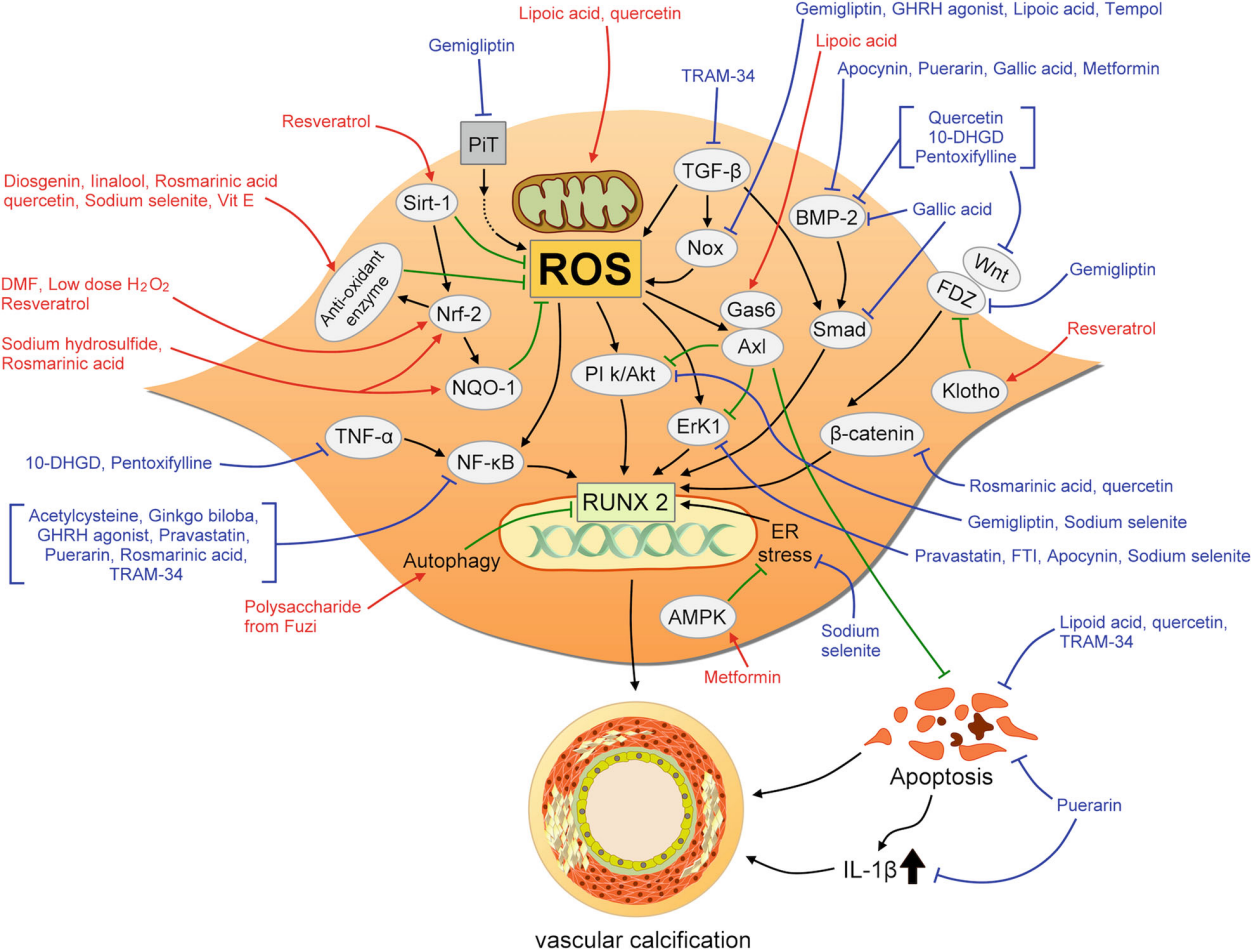
An illustrative diagram showing the plausible molecular mechanisms through which each antioxidant counteracts vascular calcification. Red arrows indicate positive influences by antioxidants, while blue connecting lines indicate their inhibitory action. 10-DHGD 10-dehydrogingerdione, ER endoplasmic reticulum, FTI farnesyl transferase inhibitor, IL interleukin, Nox NADPH oxidase, ROS reactive oxygen species, GHRH growth hormone-releasing hormone, TGF transforming growth factor, TNF tumor necrosis factor

Based on the above reports, there is a high probability that multiple edible natural antioxidants can be potential candidates for managing VC, although the doses of each agent vary widely, and the mechanisms may differ depending on agent-specific or experimental model-specific issues. We have constructed an illustrative diagram to integrate the known and confirmed molecular alterations responsible for the beneficial effects of these nutraceuticals (Fig. 3).

## Antioxidants from natural non-dietary sources

We identified five types of potential antioxidants with therapeutic effects against VC based on the literature search, including linalool^61^, lipoic acid^22–24^, rosmarinic acid^63^, fermentation broth extracts from Actinobacteria^59^, and polysaccharide from Fuzi product^60^. Two (40%) of them have ROS scavenging ability, while the other three (60%) do not (Fig. 2).

Linalool, a natural terpene alcohol inherent to multiple types of flowers, is now a common constituent of fragrance with cosmetic and non-cosmetic applications^77^. Kaur et al. disclosed that linalool could scavenge ROS in VSMCs, increase antioxidant enzyme levels, and reduce the expression of PiT-1 and RUNX2 in CKD rats^61^. Lipoic acid, produced from octanoic acid in mitochondria, is a cofactor for multiple physiological reactions and a powerful antioxidant known to date. Several groups have proposed that lipoic acid could substantially attenuate VC in vitro and in vivo. Liberman et al. showed that lipoic acid ameliorated aortic calcification and improved vascular compliance in high cholesterol- and vitamin D-fed rabbits by inhibiting Nox4 expression and reducing ROS^22,24^. Similar to quercetin, lipoic acid exhibited a beneficial effect against VC by improving mitochondrial function and inhibiting apoptosis, through restoring Gas6/Axl survival signaling^23^. Rosmarinic acid is a phenolic compound purified from medicinal plants belonging to the families *Apiaceae*, *Boraginaceae*, and *Lamiaceae* with anti-inflammatory and antioxidant properties^78^. Ji et al. showed that rosmarinic acid reduced VSMC calcification by upregulating Nrf2, NADPH quinone dehydrogenase 1 (NQO1) expression, increasing antioxidant enzyme levels while inhibiting NF-κB and β-catenin signaling in vitro and in vivo^63^. Finally, extracts of bacterial and plant products are also found to counteract VC experimentally. Preliminary results from Liao et al. showed that polysaccharide extracted from Radix *Aconiti Carmichaeli*, a raw product with anti-oxidant activity, could reduce calcification severity of ox-LDL-treated VSMCs, partially by activating autophagy^60^. Salimi et al. also found that metabolites extracted from cultured *Nocardia* strain could attenuate protein oxidation, reduce inflammation, and reduce VSMC calcification severity^59^. The potential downstream effectors of these natural non-dietary compounds are summarized in Fig. 3. Although parts of the above compounds are derived by extracting botanical and bacterial metabolites and are likely heterogeneous, these findings suggest that a plentiful of natural substances can be tested as powerful treatments for VC.

## Antioxidants of synthetic origin or pharmaceuticals

Multiple non-natural chemicals/medications also exhibit antioxidant properties and are effective against VC. We identified 19 types of such candidates with potential therapeutic effects against VC: acetylcysteine^33^, alendronate^49^, BMP inhibitor^39^, dimethyl fumarate^47^, farnesyltransferase inhibitor^41^, gemigliptin^57^, growth hormone-releasing hormone (GHRH) receptor agonist^58^, metformin^42^, pentoxifylline^64^, pravastatin^33^, pyrrolidine dithiocarbamate (PDTC)^34^, simvastatin^32^, sodium hydrosulfide^53^, sodium selenite^37,45^, STS^36,43,44^, Tempol^40^, TRAM-34^46^, topical poly(1,8-octamethylene-citrate-co-cysteine) (POCC)^54^, and hydrogen peroxide^51^. Among these, 11 (57.9%) were medications with specific clinical indications, while 8 (42.1%) were non-medicinal chemicals. Seven of them (36.8%) have ROS scavenging ability, while the other 12 (63.2%) do not (Fig. 2). We have briefly summarized the action of each agent in the following section.

STS is first such chemical being tested for its activity against VC and reaps the most attention by researchers in this field. Originally used for the treatment of cyanide toxicity, STS is also found to have strong antioxidant properties and a calcium-chelating tendency^79^. Pasch et al. showed that STS significantly reduced VC severity in uremic rats, and they proposed that STS acted by inducing acidosis and urinary excretion of calcium^36^. Subsequent studies suggested that STS could suppress BMP-2 expression and upregulate MGP in calcified VSMCs^43^, indicating that STS may interfere directly with the pathophysiology of VC in addition to its ability to bind calcium. With the assistance from adipocytes, STS may attenuate the calcification of arterial walls to a greater extent^44^.

The most renowned antioxidant medication is probably acetylcysteine, which acts through its conversion into substrates for hepatic glutathione synthesis, and is used clinically for managing acetaminophen overdose and for prophylaxis of contrast-induced kidney injury^80^. While examining the vasculopathic effect of protease inhibitors, Afonso et al. found that acetylcysteine could retard the progression of VSMC calcification through its antioxidant and anti-inflammatory capability^33^. Sodium selenite, a formula that delivers the trace element selenium to host individuals, has been tested in two studies for its anti-calcification ability, since selenium restoration has been shown to raise glutathione peroxidase activity and to attenuate ROS production in patients with different illnesses^81^. Liu et al. revealed that sodium selenite could inhibit the ERK1/2 pathway, leading to a lower severity of calcification in a model of H_2_O_2_-induced VSMC phenotypic switch^37^. They also found that sodium selenite suppresses OS by elevating glutathione peroxidase activity, inhibiting PI_3_K/Akt activity and endoplasmic reticulum stress in VSMCs^45^, supporting its use as a treatment for VC.

Other medications, including anti-osteoporotic medication (alendronate), oral hypoglycemic agent (gemigliptin and metformin), hypolipidemic agents (pravastatin and simvastatin), medication for multiple sclerosis (dimethyl fumarate), microcirculatory insufficiency (pentoxifylline), and growth hormone deficiency (GHRH receptor agonist) have all been associated with suppressed ROS production and attenuated VC in different experimental models. Cutini et al. showed that alendronate increased NO production and activated MAPK signaling pathway, while it attenuated inflammation and reduced VC severity^49^. Ha et al. discovered that dimethyl fumarate administration led to Nrf2 activation, BMP-2 downregulation, and less severe VC in a calcified VSMC model and in animals with vitamin D-induced aortic calcification^47^. Pentoxifylline is also shown to decrease the expression of BMP-2, Wnt3a, and RANK in addition to its anti-inflammatory and anti-calcific properties^64^. Gemigliptin belongs to the dipeptidyl peptidase-4 inhibitor class of antidiabetic agents, which exhibit a pleiotropic effect through glucagon-like peptide-1 (GLP-1)-dependent and -independent mechanisms including insulin sensitization and the inhibition of oxidation and apoptosis^82^. Choi et al. reported that gemigliptin attenuated VC through a variety of beneficial molecular alterations, including decreased expressions of PiT-1 and Nox4 with reduced ROS production, and suppressed PI_3_K-Akt and Wnt-FDZ pathway activities^57^. On the other hand, metformin has been shown to ameliorate VSMC calcification through AMPK-independent (BMP-2 inhibition) and AMPK-dependent (AMPK and eNOS activation) mechanisms^42^. Statins also exhibit anti-inflammatory and antioxidant properties, apart from their cholesterol-lowering effect. Ivanovski et al. first showed that simvastatin was capable of decreasing aortic OS markers and reducing aortic calcification in ApoE knockout mice with CKD, independent of cholesterol levels^32^. This is further supplemented by Afonso et al.’s work, which suggested that pravastatin could decrease local OS by inhibiting farnesyl transferase in calcified VSMCs^33^. GHRH receptor agonists, a hormonal agent not previously investigated for its vascular action, was shown to alleviate aortic calcification via NF-κB downregulation and the reduction in inflammation^58^. It seems that many of these antioxidant medications exert their actions by modulating NF-κB expression and lowering the contribution of inflammation to VC pathogenesis, although some positively influence vascular health through multiple mechanisms (Fig. 1). Judging from their efficacy against VC, it is expected that part of this medication list may eventually gain momentum as an effective therapy for VC treatment.

Several non-medicinal chemicals with antioxidant property are also found to protect against VC, serving as potential treatment candidates. It was expected that BMP inhibitors LDN-193189 would retard VC progression in LDL receptor knockout mice; pharmacological BMP inhibition additionally attenuated the production of ROS and reduced the severity of inflammation in vascular tissues, as shown by Derwall et al.^39^. The inhibition of protein prenylation, which markedly influences isoprenoid synthesis and mevalonate metabolism, using farnesyl transferase inhibitor R115777 has also been shown to reduce aortic tissue OS levels and ameliorate calcium deposition by inhibiting Ras-Raf signaling^41^. Interestingly, calcium-activated potassium channel blocker TRAM-34, which helps maintain VSMC membrane potential and affects intracellular Ca^2+^ signaling, was found to decrease VSMC calcification by inducing NO release, attenuating TGF-β signaling, and inhibiting NF-κB activation with reduced apoptosis^46^. The administration of sodium hydrosulfide, which strengthens endogenous antioxidant hydrogen sulfide action, was similarly found to reduce VC severity by decreasing H_2_O_2_ production, inhibiting TNF-α expression, and activating Nrf2-NQO1 pathway^53^. Dithiocarbamate compounds, such as PDTC, have been reported to exhibit an even stronger antioxidant property than *N*-acetylcysteine by inhibiting NF-κB activation^83^. Mody et al. showed that PDTC could substantially halt the process of calcification in vascular cells, although the mechanisms involved have not been explored^34^.

Synthetic compounds exhibiting antioxidant properties are also being used to fight VC. Poly(1,8-octamethylene citrate) (POC), an elastomer constructed as a biodegradable material, has been shown previously to decrease ROS production in neighboring tissues when coated onto vascular grafts^84^. Its derivative, POCC, when coated, was also found to attenuate local oxidized DNA and lipid damages, accompanied by a reduction in graft calcification^54^.

The confirmed effector mechanisms of these medicinal and non-medicinal chemicals with antioxidant properties against VC are summarized in an integrative way in Fig. 3.

## Antioxidants with contradictory findings regarding VC

Some of these non-medicinal compounds have shown contradictory findings in the pathogenesis of VC. For example, tempol, a frequently used experimental superoxide dismutase mimetic, has been controversial regarding its effect on the progress of VC. Yamada et al. showed that tempol could reverse the osteoblastic transdifferentiation of VSMCs and improve uremic VC in CKD rats through ROS reduction and Nox4 inhibition^40^. However, Liberman et al. showed that in a rabbit model of atherosclerotic calcification, although tempol lowered aortic ROS levels, it paradoxically increased medial calcification and plaque burden^24^. This can be attributed to the species-dependent or calcification model-dependent actions of tempol, but further studies are needed before arriving at a definite conclusion. In addition, hydrogen peroxide is traditionally deemed an endogenous OS mediator and used exogenously as a ROS inducer. Intriguingly, Zhang et al. showed that a very low concentration (0.01 mM) of hydrogen peroxide could on the contrary reduce intracellular ROS generation and stimulate Nrf2 activity, decreasing the severity of VSMC calcification in vitro without inducing apoptosis^51^. This phenomenon is reminiscent of the physiological role of “preconditioning” maneuver, which protects specific organs from severe damage if they are subjected to mild insult beforehand. Nonetheless, their findings are still preliminary and need replication.

## Clinically tested antioxidants for treatment of VC

We identified five clinical studies testing one parenteral and the other topical agent with an antioxidative ability regarding their efficacy in treating VC (Table 2)^13–17^. STS, which has been repeatedly tested in experimental models for inhibiting VC progression, is also used clinically to treat calcification involving arteries from different anatomical locations. However, it is evident that these studies are anecdotal, enrolling few patients, and the results appear heterogeneous with some showing calcification regression^14,15^ while others reporting no interval changes^13,16^. A topical chemical, cerium nitrate, has been shown to improve the overall outcome of patients with calciphylaxis when used for wound dressing, presumably through ROS scavenging and chelating local calcium^17^. Further studies are still needed to confirm these findings.

**Table 2:** Clinical studies investigating the therapeutic effect of anti-oxidants in patients with clinical VC

Medications	Sample size	doses	Disease type	Response	Reference
Sodium thiosulfate	22 HD	25 g thrice weekly IV for 3 months	Carotid, coronary, and aortic calcification	Mean annual VC changes did not increase	^13^
Sodium thiosulfate	6 HD	10 g thrice weekly in dialysate for 6 months	Coronary calcification	66% had calcification reduction vs. 0% in control	^14^
Sodium thiosulfate	18 HD	10 g thrice weekly IV for 6 months	Abdominal aortic calcification	Modest calcification reduction but moderate leg pain improvement	^15^
Sodium thiosulfate	38 HD	0.18 g/kg thrice weekly IV for 3 months	Coronary calcification	Treatment group had stable calcification while control group progressed	^16^
Cerium nitrate	71	Topical application to wound	Calciphylaxis	OR 0.44 (0.2–0.99) for mortality	^17^

*HD* hemodialysis, *IV* intravenous, *OR* odds ratio, *VC* vascular calcification

Finally, we undertook a comprehensive search in the clinicaltrials.gov registry to look for any completed or ongoing clinical trials involving the use of these 36 antioxidative compounds for treating VC, up to 7 June 2019. Only two registered trials attempted to test these agents against the target phenotype, VC, including alendronate and statin, although the results have not been available. It is anticipated that more antioxidative compounds will gain momentum as anti-VC medications in the future.

## Conclusion and future perspective

Natural compounds are rich sources of potential therapeutic candidates for various diseases, including neurodegenerative disorders, metabolic dysregulation, cardiovascular diseases^85^, and VC. Repurposing the existing medications for alternative applications is an important way of identifying novel therapies with known safety profiles^86^. For VC, the pivotal components in its pathogenesis are ROS production and inflammation^87,88^, which are targeted by many natural compounds and existing medications. Moreover, foods rich in these natural compounds may also be beneficial for patients with VC, although the ingestion amount needed to achieve the optimal result remains elusive. With the knowledge gained by the current literature, we expect that more antioxidants will emerge in the future as potential candidates in the therapeutic armamentarium against VC.

## Data Availability

Literature summary data will be available upon reasonable request.
